# Enhanced Performance of Bioelectrodes Made with Amination-Modified Glucose Oxidase Immobilized on Carboxyl-Functionalized Ordered Mesoporous Carbon

**DOI:** 10.3390/nano11113086

**Published:** 2021-11-16

**Authors:** Chuhan Lv, Xuewei Yang, Zongkang Wang, Ming Ying, Qingguo Han, Shuangfei Li

**Affiliations:** 1 Guangdong Technology Research Center for Marine Algal Bioengineering, Guangdong Key Laboratory of Plant Epigenetics, College of Life Sciences and Oceanography, Shenzhen University, Shenzhen 518060, China; LVCHU0510@163.com (C.L.); yingming@szu.edu.cn (M.Y.); hanqingguoszu@163.com (Q.H.); 2 Shenzhen Batian Ecological Engineering Co., Ltd., Shenzhen 518055, China; wangzongkang712@163.com

**Keywords:** carboxylation, chemical amination, glucose oxidase

## Abstract

This research reveals the improved performance of bioelectrodes made with amination-modified glucose oxidase (GOx-NH_2_) and carboxyl-functionalized mesoporous carbon (OMC-COOH). Results showed that when applied with 10 mM EDC amination, the functional groups of NH_2_ were successfully added to GOx, according to the analysis of ^1^H-NMR, elemental composition, and FTIR spectra. Moreover, after the aminated modification, increased enzyme immobilization (124.01 ± 1.49 mg GOx-NH_2_/g OMC-COOH; 2.77-fold increase) and enzyme activity (1.17-fold increase) were achieved, compared with those of non-modified GOx. Electrochemical analysis showed that aminated modification enhanced the peak current intensity of Nafion/GOx-NH_2_/OMC-COOH (1.32-fold increase), with increases in the charge transfer coefficient *α* (0.54), the apparent electron transfer rate constant *k_s_* (2.54 s^−1^), and the surface coverage *Γ* (2.91 × 10^−9^ mol·cm^−2^). Results showed that GOx-NH_2_/OMC-COOH exhibited impressive electro-activity and a favorable anodic reaction.

## 1. Introduction

The combination of nanomaterials and proteins (e.g., enzymes, antibodies, etc.) has been widely used in biosensors, biocatalysis, bioelectronics, biofuels, drug delivery systems, and biomedicine for a long time [1,2]. In order to achieve these applications, they are usually functionalized by various methods or incorporated into a polymer matrix [3,4,5] in order to ensure that the immobilized biologically active ingredients have better stability and functional density [6]. In particular, improving the interface reaction between the biomolecules and the electrode surface can be considered to be one of the most popular applications of nanomaterials [5], because it is the basis for the construction of biosensors, biomedicines, and other bioelectrochemical systems. The electron transfer of proteins is mainly controlled by three factors [7]: reorganization energies; potential differences and orientations of involved redox-active sites; and distances between redox-active sites and mediators. The effective kinetic barrier for electron transfer is the protein or glycoprotein shell surrounding the active site [8]. Since carbon nanomaterials can promote direct electronic communication between the redox sites of proteins and electrodes, they have become attractive materials for direct electrochemistry of enzymes in bioelectrochemistry [8,9,10]. Thus, how to improve catalyst efficiency and promote electron transfer between the enzyme redox center and the electrode [6], so as to obtain a biosensor with good performance, merits further exploration [9].

GOx is widely used in the construction of glucose sensors, owing to its high selectivity to glucose and high activity in a wide pH range [11]. There are amino, carboxyl, and hydroxyl groups in the amino acid part of the enzyme, which are used for covalent bonding in the electrode material [12]. The strength of the enzyme-supported multipoint covalent reaction strongly depends on the number of reactive groups [13]. It has been shown that enriching the enzyme surface on the amino surface via different techniques can successfully enhance the process of multipoint covalent immobilization [14]. The purposes of chemical functionalization are as follows [15]: (1) owing to appropriate interactions by surface anchoring specifically, preferentially adsorbed or grafted enzymes produce carbon nanomaterials close to the active site of GOx, improving the electronic transport capacity of the electrode because it provides a good electron-conductive network [2,8,9,10]; and (2) to increase the load of enzymes, thereby increasing the catalytic current and, finally, enhancing the amount of electroactive enzymes. Moreover, charged functional groups exposed on the nanomaterials’ surface can control or guide the protein adsorption [16] through group types [17], hydrophobicity [18], and particle surface charge [19]. Gloria Fernandez-Lorente et al. [20] chemically aminated the lipase of *Bacillus thermocatenulatus* (BTL_2_), which was covalently immobilized on highly activated acetaldehyde–agarose; the immobilization rate of aminase was higher than that of the natural enzyme at pH 10, and the stability of the immobilized enzyme was improved. Maryam Ashjari et al. [21] chemically aminated *Rhizopus oryzae* lipase (ROL) and covalently attached it to epoxy-functionalized silica and silica nanoparticles (MCM-41 and SBA-15); the immobilized derivatives of aminated ROL had higher thermal stability and co-solvent stability, and the selectivity and reusability of ROL were greatly improved. Rafael C. Rodrigues et al. [22] covalently linked the lipase of aminated thermophilic mold (TLL) to glyoxylic acid agarose at multiple points and compared it with the unmodified enzyme; the stability of the immobilization of the chemically modified enzyme during the heat inactivation process was improved fivefold. At present, there are many reports on the chemical amination and immobilization of GOx [20,21,22], but there has only been one report of an electrochemical sensor with chemical amination of laccase-immobilized and -functionalized carbon nanotubes for phenol detection [23]. Moreover, there are few reports on the electrochemical performance of aminated GOx-immobilized functional nanocarbon materials.

For enzymatic bioelectrodes, the immobilization carrier material greatly affects the efficiency of the fuel cell via electrical conductivity and stability range. It has been reported that when using a porous electrode in place of a planar electrode, the power density was increased by 72%, with enhanced effective area and improved material transport characteristics [24]. Compared with other electrode materials, carbon-based materials (such as graphite fiber brush [25], reticulated vitreous carbon (RVC) [26], carbon cloth [27], carbon felt [28], and graphite rods [29]) are more broadly used because of their valuable properties, such as the possibility of chemical surface modification, high electrical conductivity, high biocompatibility, and low prices [30]. Moreover, nanocarbon materials have been successfully used in various electrochemical biosensing schemes, and have shown excellent application prospects in designing new sensing systems and enhancing the performance of biological analysis and detection [31]. Therefore, carbon-based porous nanomaterials such as ordered mesoporous carbon (OMC) have been pursued as promising electrode materials due to their unique properties of small size, large surface area, high conductivity, and high chemical and thermal stability [32]. To improve the effective electron transfer rate, surface modification was applied to arrange the correct electron transfer direction so that the electrode surface was sufficiently close to the center of each active substance [2,9,30]. Functionalizing the surface of nanomaterials according to the charge groups (such as –COOH, –NH_2_, etc.) is also conducive to the immobilization of enzymes and the driving force of adsorption (the electrostatic interaction, hydrophobic interaction, or hydrogen bond interaction) [17,33,34,35,36,37,38,39].

## 2. Materials and Methods

### 2.1. OMC Carboxyl Functionalization

OMC functionalization was performed as described in a previous work [40]. In this oxidation experiment, 1 g of OMC (pore volume of 0.5 cm^3^·g^−1^, surface area of 150–250 m^2^·g^−1^, 699640; Sigma-Aldrich, Shanghai, China) was added to 30 mL of the APS solution (2 M ammonium persulfate ((NH_4_)_2_S_2_O_8_, APS) in 2 M sulfuric acid (H_2_SO_4_)), stirring at 30 rpm for 24 h at 50 °C (utilizing a DF-101S heat-collecting magnetic stirrer (AKΛZAN, Shanghai, China)). The obtained OMC-COOH was filtered and washed 5 times, and then vacuum-dried overnight at 60 °C.

### 2.2. Chemical Amination of Enzymes

As described in [23], GOx (246 U·mg^−1^, G8032, Solarbio, Beijing, China) was aminated. In short, a combination of 25.0 mL of non-modified GOx solution (4 mg/mL) with 25 mL of 1.0 M ethylenediamine (EDA) solution and 50 mM N-(3-dimethylaminopropyl)-N′-ethylcarbodiimide hydrochloride (EDC) was mixed for 1.5 h at pH 4.75, and then dialyzed 5 times with distilled water at 4 °C using an ultrafiltration tube (molecular weight cutoff of 50 kDa, Thermo Scientific, Waltham, MA, USA). The aminated GOx (GOx-NH_2_) was stored at −80 °C, dried under vacuum using a lyophilizer (triad 2.51, LABCONCO, south Kansas City, MO, USA), and stored at −20 °C for further use.

### 2.3. Enzyme Immobilization on OMC-COOH

The immobilization test was carried out by suspending 20 mg of OMC-COOH in 0.5 mL of 0.1 M pH 7.0 PBS buffer containing 10 mg·mL^−1^ of unmodified GOx or aminated GOx (GOx-NH_2_) in the centrifuge tube. Then, the mixture was incubated at 10 °C with stirring at 220 rpm (LYZ-D2403 superimposed shaker, Longyue, Shanghai, China) for 6 h to reach adsorption equilibrium [41]. After fixation, the enzyme-immobilized materials were centrifuged (75008800 Medifuge Centrifuge, Thermo, Waltham, MA, USA) at 10,000 rpm for 5 min. The supernatant was then removed, dried under vacuum using a lyophilizer (triad 2.51, LABCONCO, south Kansas City, MO, USA), and stored at −20 °C for further use. In each set of experiments, three biological replicates were performed.

The quantity of unmodified GOx or aminated GOx immobilized on OMC-COOH particles was determined through the original concentration and terminal concentration of the enzyme via UV spectrophotometry at 450 nm (EPOCH 2 microplate reader, BioTek, Vermont, USA). The glucose oxidase activity of the enzyme-immobilized composite materials (GOx/OMC-COOH and GOx-NH_2_/OMC-COOH) was detected using a glucose oxidase assay kit (BC0695, Solarbio, Beijing, China). In each set of experiments, three biological replicates were performed.

### 2.4. Inactivation of GOx/OMC-COOH and GOx-NH_2_/OMC-COOH

The immobilized enzyme samples obtained after lyophilization were each immersed in 0.1 M pH 7.0 PBS buffer and placed in a water bath at 4 °C, 10 °C, 25 °C, 37 °C, or 50 °C for 2 h to measure the effect of temperature on their activity. Then, samples were immersed in 0.1 M pH 4.4, 5.4, 6.4, 7.4, or 8.4 PBS buffer solution and incubated in a water bath at 25 °C for 2 h, and the effect of pH on their activity was measured. A single obtained immobilized enzyme sample was immersed in 0.1 M pH 7.4 PBS buffer solution and incubated at 25 °C for 6 h at room temperature to measure the effect of time (0, 0.5, 1, 1.5, 2, 4, 6 h) on its activity. In each set of experiments, three biological replicates were performed.

### 2.5. Fabrication of GOx/OMC-COOH and GOx-NH_2_/OMC-COOH Bioelectrodes

The enzyme bioelectrode was made by sonicating 2 mg of GOx/OMC-COOH and GOx-NH_2_/OMC-COOH with 150 μL of pH 7.0 PBS buffer solution (containing 6 μL of 0.5% Nafion) for 5 min and pipetting a 70 μL dispersion onto the carbon cloth as the anode [1]. The carbon cloth electrodes coated with Nafion/GOx/OMC-COOH and Nafion/GOx-NH_2_/OMC-COOH were placed in a fume hood for 4 h, then rinsed carefully with pH 7.0 PBS buffer and stored at 4 °C before use.

The elemental composition of the C, H, and N contents of the GOx and GOx-NH_2_ were determined using an organic element analyzer (Vario MICRO Cube, Elementar, Germany). The N–H spectra of GOx and GOx-NH_2_ were measured using a nuclear magnetic resonance instrument (AVANCEIII 400MHz, Bruker, Switzerland) to explore the change in their chemical shift. Fourier-transform infrared spectroscopy (FTIR, Nicolet 6700, Nicolet, Thermo Fisher Scientific, Waltham, MA, USA) was used to determine the changes in the functional groups of GOx and GOx-NH_2_; the measurement range was 400–4000 cm^−1^. Electrochemical experiments were performed at room temperature in PBS buffer in the absence and presence of glucose. The impedance EIS was measured at 5 mM K_3_[Fe(CN)_6_]/K_4_[Fe(CN)_6_] in 0.1 M KCl solution (frequency range: 10^−2^–10^−5^ Hz) [42].

## 3. Results

### 3.1. The Effect of Amination on Enzyme Immobilization

**Figure 1:**
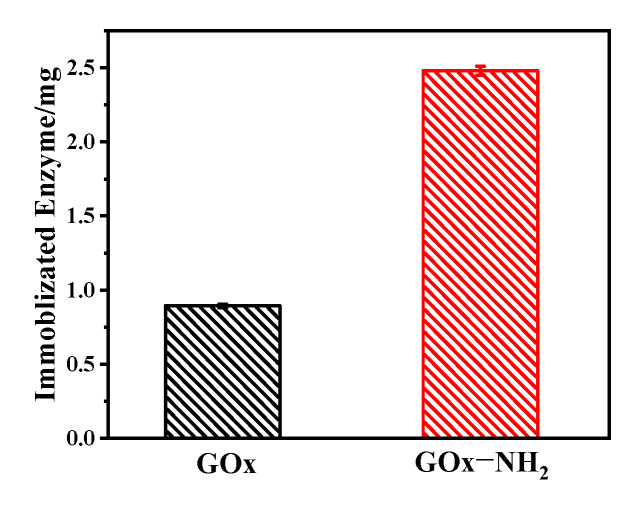
Adsorption capacity of unmodified GOx (black) and aminated GOx (red) on OMC-COOH.

In order to determine the influence of the amino group on the enzyme surface, an adsorption study was carried out and the concentration of the supernatant was measured in the solution before and after the adsorption equilibrium to quantify the adsorption capacity (Figure 1). This revealed an interesting correlation between the porous network of the carbon materials and the immobilization of the enzyme. In this experiment, the loading amount of the aminated GOx (GOx-NH_2_) was 2.48 mg of enzyme/20 mg of OMC-COOH. It was observed that the amount of aminated GOx adsorbed on OMC-COOH was 2.77 times higher than that of unmodified GOx on OMC-COOH. These results indicated that when increasing the protein-supported multipoint attachment with aminated groups [43], the protein was fixed more on the OMC-COOH. Gloria Fernandez-Lorente et al. [20] chemically aminated the lipase from *Bacillus thermocatenulatus*, which can be fixed more easily on glyoxyl–agarose, and is more stable than natural enzymes. Maryam Ashjari et al. [21] chemically aminated *Rhizopus oryzae* lipase; compared with the non-aminated enzyme, it was able to immobilize a higher number of enzymes on epoxy-functionalized silica and silica nanoparticles. Wilson et al. used a chemical amination method to enrich the surface amino groups of *Geotrichum candidum* lipase and immobilize it on a carrier based on carboxymethyl and sulfopropyl agarose-based supports, which increased fish oil hydrolysis by 2.40-fold and 3.20-fold, respectively [44].

The free GOx-NH_2_ kept 87.23% of its activity compared to the free unmodified GOx, indicating that after the chemical reactions to add the aminated groups, the glucose oxidase maintained its activity well. Since the ion bridge may be slightly destroyed and changed by the repulsive force, the modification can decrease the activity of the enzyme to a certain extent [45]. In addition, Table 1 shows that although chemical amination made the unit enzyme activity low, the total enzyme amount adsorbed by the material increased significantly—the total enzyme amount of GOx-NH_2_ immobilized on OMC-COOH was 1.77 times greater than that of GOx immobilization. This shows that the total enzyme activity of GOx-NH_2_/OMC-COOH is relatively high (after calculation, the significant difference *p* = 0.040, which is less than 0.050, so the enzyme activity of the two enzyme–carbon composite materials is significantly different). As mentioned above, amination may increase the chemical reactivity by enriching the amount of immobilized enzyme [46], thereby increasing the enzyme activity of the enzyme-containing composite material.

### 3.2. Characterization of Aminase

#### 3.2.1. ^1^H NMR Spectral Analysis

**Figure 2:**
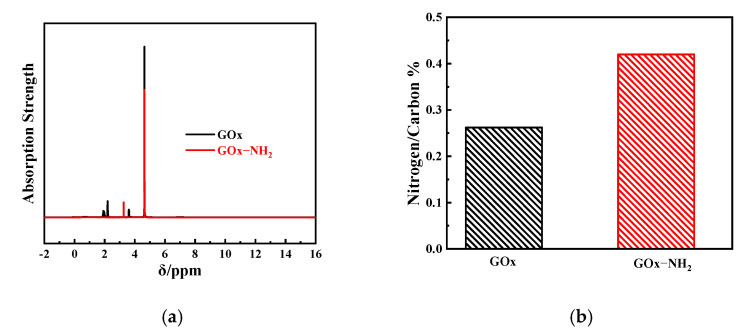
(**a**) ^1^D ^1^H NMR spectra of unmodified GOx (black) and aminated GOx (red). (**b**) Elemental analysis results of GOx (black) and aminated GOx (red).

The ^1^H NMR spectra of the enzymes with unmodified GOx and aminated GOx (Figure 1) allowed the assignment of the H-5 protons in the samples. H-5 in samples with carboxylic acid groups (the ester groups) was found around 4.8 ppm [47]. The signal at 3.8 ppm corresponded exclusively to the anomeric protons, H-1_OH_ (Figure 2; black line) [47], while the other protons represented by the red line in Figure 2 appeared as new signals at ~3.3 ppm, which may have been caused by the amination reaction. Tightly bound ligand molecules alter the binding site on the protein, and the chemical shifts in the protein resonances typically exhibit the largest chemical shift changes. The result of ^1^H NMR analysis indicated that the aminated groups were successfully added to GOx.

#### 3.2.2. Element Composition Analysis

The results of elemental analysis are listed in Figure 2b. The hydrocarbon ratio (N/C) of aminated GOx was significantly higher than that of unmodified GOx, indicating the successful addition of NH_2_ to the glucose oxidase. Solid EDC was added to the suspension at 10 mM as a final concentration to ensure that all exposed carboxyl groups of the protein were completely modified [20,48]. The increase in the ratio of nitrogen to carbon could be the result of enzyme amination.

#### 3.2.3. Fourier-Transform Infrared (FTIR) Spectrum Analysis

**Figure 3:**
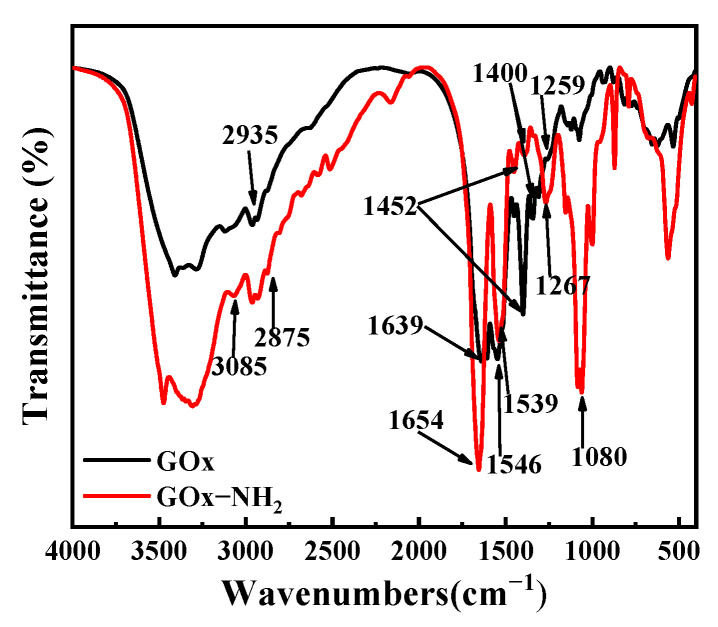
Fourier-transform infrared (FTIR) spectra of unmodified GOx and GOx-NH_2_.

The FTIR spectrum results of GOx and GOx-NH_2_ can be seen in Figure 3. The peak near 3085 cm^−1^ in the spectra of GOx and GOx-NH_2_ belongs to asymmetric CH_3_ stretching, the peak at 2935 cm^−1^ belongs to asymmetric CH_2_ stretching, the peak at 2875 cm^−1^ belongs to symmetric CH_3_ stretching, and the peak at 1080 cm^−1^ can be attributed to the stretching of C–O [49,50]. The bands of the amide I and II peaks of GOx are located at 1639 cm^−1^ and 1546 cm^−1^, respectively, while the bands of the amide I and II peaks of GOx-NH_2_ are located at 1654 cm^−1^ and 1539 cm^−1^, respectively [51,52]. The amide III peak is also located near 1350–1200 cm^−1^ [51], and the bands of the amide III peaks of GOx and GOx-NH_2_ are located at 1259 cm^−1^ and 1267 cm^−1^, respectively. At 1452 cm^−1^ and 1400 cm^−1^, is the peaks are attributed to the shear of the side-chain CH_2_ and the tensile vibration of C–O in =CO, respectively [49,53]. The peak at 3290 cm^−1^ can be attributed to the N-H vibration of glucose oxidase [53,54]. In the results of the spectral analysis, although some peak positions were shifted, the peak intensity and peak area of the amide in the GOx-NH_2_ spectrum were stronger than those of GOx, clearly showing that the aminase treatment increases the content of –NH_2_ [43].

### 3.3. The Effect of Amination on Enzyme Immobilization

#### 3.3.1. Temperature

**Figure 4:**
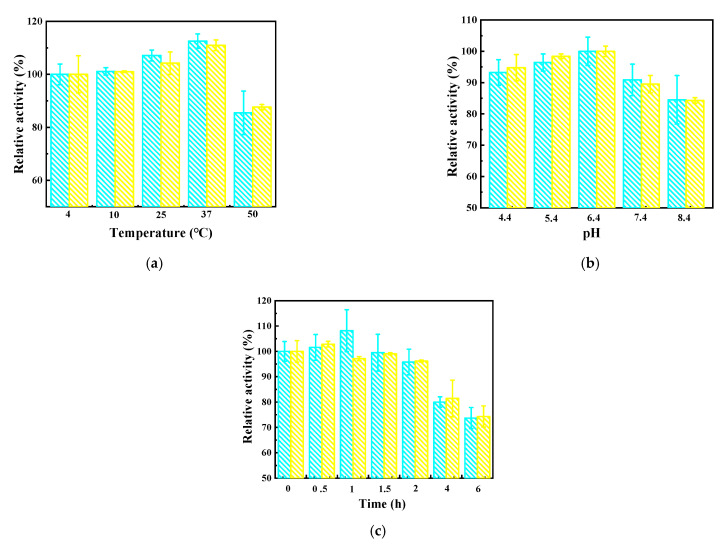
The effects of temperature (**a**), buffer pH (**b**), and time (**c**) on the enzyme activities of GOx/OMC-COOH (blue) and GOx-NH_2_/OMC-COOH (yellow).

For the GOx and GOx-NH_2_ immobilized on the OMC-COOH, the modification by amination slightly changed the enzyme activity (Figure 4a). The activity of the unmodified and aminated glucose oxidase at 4 °C and 10 °C was almost the same, but it gradually increased with the increase in the reaction temperature, showing the maximum enzyme activity at 37 °C, which is the normal body temperature. This indicates that the GOx-NH_2_ immobilized on the OMC-COOH possesses great potential and advantages for work involving the physiological fluids of the human body. When the temperature was increased by 50 °C, the activity of the immobilized enzyme decreased, but the activity of the immobilized enzyme after amination was higher than that of the untreated immobilized enzyme, indicating that modification via amination could improve the tolerance of glucose oxidase to high temperatures.

#### 3.3.2. Buffer pH

The effect of pH on the stability of GOx and GOx-NH_2_ was determined by incubating the same amounts of the enzymes in 0.1 M pH 4.4, 5.4, 6.4, 7.4, and 8.4 PBS buffer at 25 °C for 2 h (Figure 4b). The optimal pH value of immobilized GOx and GOx-NH_2_ was 6.4. When the pH was 7.4, the immobilized GOx and GOx-NH_2_ retained 90.87% and 89.53% of the enzyme activity, respectively. Even when the pH increased to 8.4, the enzyme activity still retained 84.52% and 84.29% of the activity of immobilized GOx and GOx-NH_2_, respectively. Both the immobilized GOx and GOx-NH_2_ showed excellent stability in the measured pH range (4.4–8.4).

#### 3.3.3. Time

At room temperature (25 °C), the enzyme activity of the GOx and GOx-NH_2_ on OMC-COOH was measured over the course of 6 h (Figure 4c). The results showed that GOx and GOx-NH_2_ possessed good stability at 0–2 h, and retained 96.19% and 95.79% of their initial activity, respectively, at 2 h. As the time went by, the activities of GOx/OMC-COOH and GOx-NH_2_/OMC-COOH decreased slowly. After 6 h, the residual activities of immobilized GOx and GOx-NH_2_ were reduced to 73.68% and 74.29%, respectively. It was observed that the activity of the immobilized enzyme after amination was higher than that of the untreated immobilized enzyme, indicating that the stability of the modified enzyme is slightly higher.

### 3.4. Direct Electrochemistry of Nafion/GOx/OMC-COOH and Nafion/GOx-NH_2_/OMC-COOH Bioelectrodes

**Figure 5:**
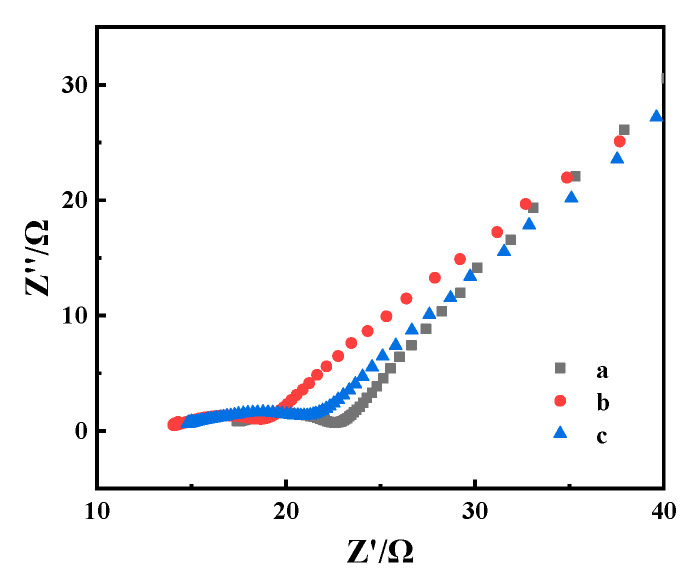
Nyquist plots (−Z″ vs. Z′) of Nafion/OMC-COOH (a), Nafion/GOx/OMC-COOH (b), and Nafion/GOx-NH_2_/OMC-COOH (c).

The Nyquist plot of the impedance spectrum includes a linear portion at low frequency and a semicircle portion at high frequency [11]; the former corresponds to the diffusion process, and the latter corresponds to the charge-transfer-limited process [11]. The electron transfer resistance (R_ct_) at the electrode surface is equal to the diameter of the semicircle, which can be used to describe the interface characteristics of the electrode [11,55]. Figure 5 shows the impedance spectra—represented as Nyquist plots (Z” versus Z’)—of electrodes modified with GOx/OMC-COOH and GOx-NH_2_/OMC-COOH in equimolar K_3_[Fe(CN)_6_]/K_4_[Fe(CN)_6_] solution. After immobilization of GOx or GOx-NH_2_ onto OMC-COOH, the diameter of the high-frequency semicircle increased further (b and c in Figure 5). The R_ct_ diminutions of the enzyme electrodes were in the following order: GOx-NH_2_/OMC-COOH≈GOx/OMC-COOH > bare OMC-COOH. The observed increase in diameter was due to the GOx or GOx-NH_2_ layer acting as a barrier to the charge transfer between the redox probes and the electrode surface in the solution [56]. The non-conductive properties of unmodified GOx or GOx-NH_2_ hinder the diffusion of the redox couple to the electrode surface, thereby reducing the electron transfer. Meanwhile, the decline in the slope of straight lines substantiates the successful fixation of unmodified GOx or GOx-NH_2_ on the bionanohybrid composite, which slightly reduces the diffusion process of the matrix [55]. These results indicate that unmodified GOx or GOx-NH_2_ was steadily immobilized onto the OMC-COOH surface [57].

**Figure 6:**
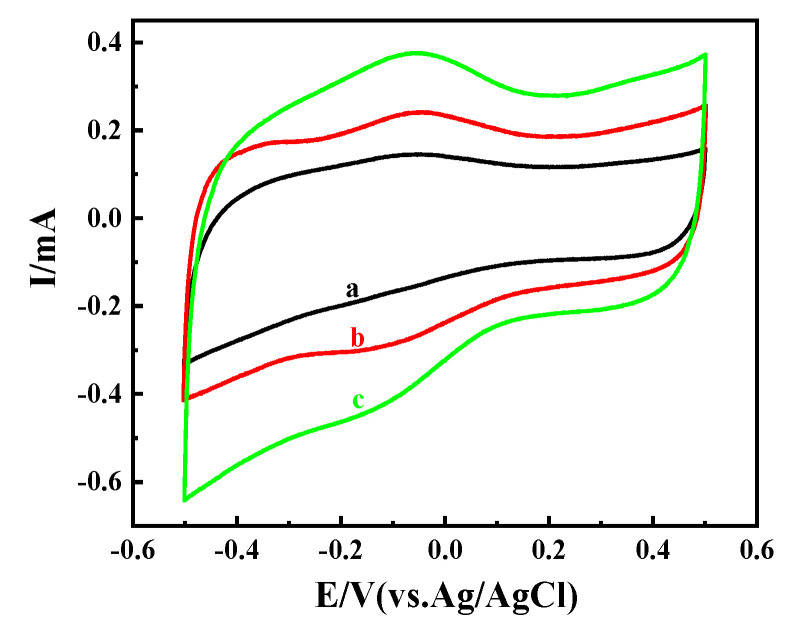
CVs (50 mV·s^−1^) of Nafion/OMC-COOH (a), Nafion/GOx/OMC-COOH (b), and Nafion/GOx-NH_2_/OMC-COOH (c) in air-saturated PBS (0.1 M pH 7.4) without glucose.

Under the same conditions, without the existence of the enzyme, naked OMC-COOH (a in Figure 6) scarcely reveals any peak values within the measurement range, which indicates that OMC and Nafion are inert; thus, the generation of current is completely dependent on the activity and amount of immobilized non-modified GOx or GOx-NH_2_ [58]. However, the redox signal increased as a result of modifying the electrode surface, in the following order: Nafion/GOx-NH_2_/OMC-COOH > Nafion/GOx/OMC-COOH > Nafion/OMC-COOH. As shown in b and c in Figure 6, the peak separation (ΔE_p_) of the bioanode was b for Nafion/GOx/OMC-COOH and Nafion/GOx-NH_2_/OMC-COOH, at scan rate of 50 mV·s^−1^, exhibiting a fast electron transfer process. Thus, a direct electron transfer of GOx or GOx-NH_2_ in the nanocomposite electrodes was achieved via the successful fixation of the enzyme on the surface of OMC-COOH [59]. Compared with the bare electrode, the oxidation current increased as the fixed amount of unmodified GOx or GOx-NH_2_ on the surface of the electrode increased. This shows that the redox and electrocatalytic activity of the functionalized enzyme on the electrode surface creates more active sites for catalysis of the redox reaction. This result shows that with the presence of the NH_2_ group, the surface of Nafion/GOx-NH_2_/OMC-COOH shows a significant advantage in terms of effective electron transfer efficiency, and builds a conductive and biocompatible microenvironment, which might play an important role in the appropriate enzyme orientation and promote the electron transfer efficiency between aminated GOx and the OMC-COOH electrode [60].

**Figure 7:**
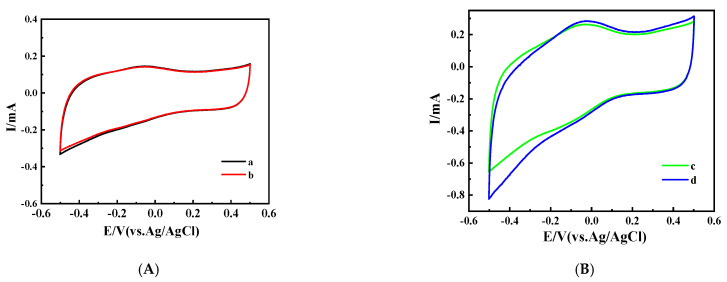

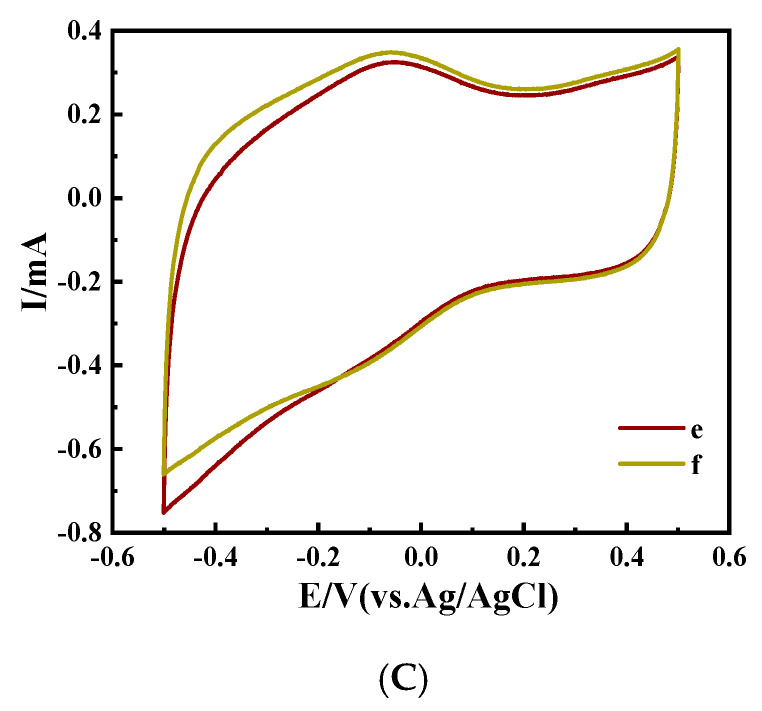
CVs (50 mV·s^−1^) of Nafion/OMC-COOH (**A**), Nafion/GOx/OMC-COOH (**B**), and Nafion/GOx-NH_2_/OMC-COOH (**C**) in air-saturated PBS 0.1 M pH 7.4 without (a,c,e, respectively)/with (b,d,f, respectively) the addition of 20 mM glucose.

The cyclic voltammogram performed on the bare OMC-COOH electrode, in the absence and presence of glucose, is shown in Figure 7A, while those of Nafion/GOxOMC-COOH and Nafion/GOx-NH_2_/OMC-COOH are shown in Figure 7B,C, respectively. In the absence and presence of glucose, as shown in Figure 7A, there was no obvious peak corresponding to the glucose oxidation in the electrode Nafion/COOH-OMC (a,b), indicating that there was no biochemical reaction on the bare COOH-OMC to produce electrons [58]. Thus, the currents observed in the Nafion/GOx/OMC-COOH and Nafion/GOx-NH_2_/OMC-COOH bioelectrodes were mainly contributed by the GOx catalysis. As Figure 7C (e,f) shows, the oxidation peak current of Nafion/GOx-NH_2_/OMC-COOH was much higher (1.21 times) than that of Nafion/GOxOMC-COOH without glucose (Figure 7B (c,f)), suggesting that it had better electrocatalytic activity. This relative increase indicates that amination could help to improve enzyme attachment onto the electrode surface.

**Figure 8:**
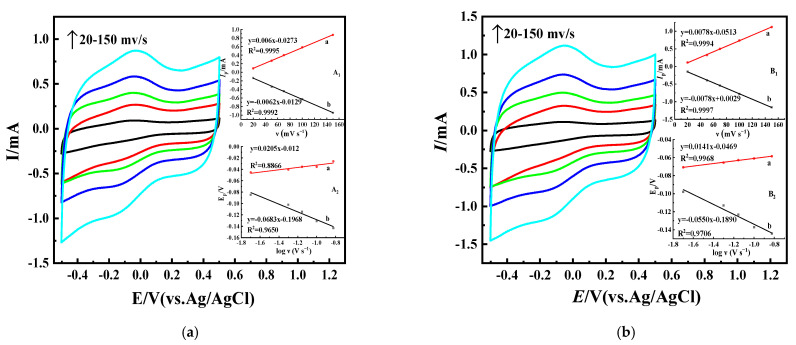
(**a**) CVs of Nafion/GOx/OMC-COOH at different scan rates (20–150 mV·s^−1^); insets (A_1_ and A_2_) are plots of anodic and cathodic peak currents vs. scan rate and peak potentials vs. log-scan rates, respectively. (**b**) CVs of Nafion/GOx-NH_2_/OMC-COOH at different scan rates (20–200 mV·s^−1^); insets (B_1_ and B_2_) are plots of anodic and cathodic peak currents vs. scan rate and peak potentials vs. log-scan rates, respectively.

The CVs of Nafion/GOx/OMC-COOH and Nafion/GOx-NH_2_/OMC-COOH at different scan rates (20–150 mV·s^−1^) in PBS buffer are also shown in Figure 8. The anodic and cathodic peak currents of the Nafion/GOx/OMC-COOH and Nafion/GOx-NH_2_/OMC-COOH redox system increased linearly with an increase in scan rates. As shown in Figure 8 (A_1_ and B_1_), the peak currents had a linear relationship with the square root of the scan rate, indicating that diffusion limitations occur via the electrode layer [61], which contributes to the surface processes of Nafion/GOx/OMC-COOH and Nafion/GOx-NH_2_/OMC-COOH. For applications of nano-composite carbon nanotube–platinum carbon nanoparticles in glucose biosensing [62], similar electrochemical behavior was also observed.

As shown in Figure 8 (A_2_ and B_2_), peak currents (i_pa_ and i_pc_) and peak potentials (E_pa_ and E_pc_) were plotted against v. A line starting from the origin (i_p_ vs. v plot) illustrates that the adsorption of enzymes on biological anodes controls the phenomenon of electron transfer [63]. Through the Laviron theory and its equation [64], the charge transfer coefficient, α, and apparent electron transfer rate constant, *k_s_* can be calculated:

*k_s_ = mnνF/RT* (1)

where *n* is the number of transferred electrons (*n* = 2), *ν* is the scanning rate (50 mV∙s^−1^), *R* is the universal gas constant (8.314 J∙mol^−1^∙K^−1^), *T* is the room temperature (298.15 K), and *m* is a constant related to peak potential separation ΔE_p_ (m is 0.69 in this work, since the nΔE_p_ value is ~47.52 mV) [64]. The charge transfer coefficients *α* of Nafion/GOx/OMC-COOH and Nafion/GOx-NH_2_/OMC-COOH are 0.41 and 0.54, respectively. The α increases as the number of immobilized enzymes increases, starting from 0.41 (Nafion/GOx/OMC-COOH) up to approximately 0.54 (Nafion/GOx-NH_2_/OMC-COOH) [65]. The calculated *k_s_* of Nafion/GOx-NH_2_/OMC-COOH was 2.54 s^−1^ which is much larger than the rate constant of GOx adsorbed on the OMC-COOH electrode (1.83 s^−1^). These results further suggest that the enzyme ammoniation can promote the electron exchange between the active site of GOx and the electrode [58]. This finding implies that the anodic reaction (GOx oxidation) is preferable.

## 4. Discussion

### 4.1. Effects of GOx Amination

Our results indicate that chemical amination of enzymes can significantly affect the immobilization and activity of GOx on OMC-COOH. The activity of immobilized aminated GOx modified with 10 mM DEC increased by 16.58% (as shown in Figure 1). The driving force of the immobilization of aminated GOx on OMC-COOH is not the reactivity of a single residue, but the density of reactive groups on the protein surface (amination can change the number of amino groups on the protein surface) [66]. The results show that the chemical amination of enzymes can significantly affect the quantity of non-modified GOx and aminated GOx fixed on OMC-COOH. The modification enriches the enzyme surface with low-pK amino groups [44,67], making the enzyme more reactive against OMC-COOH, and allowing the enzymes to be fixed on OMC-COOH at pH of 7 [14,20]. The number of highly reactive amino groups can also promote immobilization, depending on the density of the reactive groups of the support and the enzyme [66]. Therefore, amination of the enzyme slightly changes the properties of the enzyme, but greatly improves the immobilization [22].

### 4.2. Improved Characteristics of the Bioelectrode

OMCs are ideal substrates for the deposition of biomolecules because of their large surface area, high aspect ratio, and adjustable specificity of enzyme adsorption [68]. The OMCs were previously oxidized to introduce carboxylic groups on their defect sites in order to ensure their efficient adsorption to the enzyme molecule [23]. EDA was used to increase the number of primary amino groups on the GOx surface, thereby increasing the reaction between the surface amino groups produced by amination and the carboxyl groups produced by the oxidation of OMC to improve the efficiency of fixation [69]. A part of the OMC-COOH structure extends in a radial manner, which can increase the specific surface area of the composite material and improve its mechanical strength [70]. This structural optimization can effectively enhance the electron transfer between the enzyme protein structure and the electrode [69,71]. The result indicates the superiority of Nafion/GOx-NH_2_/OMC-COOH electrodes over Nafion/GOx/OMC-COOH electrodes. This performance is due to the coupling between the carboxyl group of carboxylated OMC and the amino group of aminated GOx, while retaining the structure of GOx and its catalytic effect [23]. Meanwhile, the reason that GOx can perform effective electron transfer on carbon nanomaterial electrodes is possibly because part of the protein conformational rearrangement occurs during the electron transfer process [5,8]. The changes caused by this adsorption [5,72] may be due to the conformational changes of GOx in the microenvironment that lead to the active site of GOx being close to the electrode [8].

In addition, the integration of the CV peak can calculate the total charge (Q) of the electrode, which is used to evaluate the electrical activity of the immobilized enzyme. The value of *Γ* is calculated by the formula *Γ* = *Q/nFA*, where *Γ* is the surface coverage of the enzyme electrode, *Q* is the total charge of the electrode, *F* is the Faraday constant, *n* is the number of electrons transferred, and *A* is the electrode area (1 cm^2^) [73]. The surface coverage (*Γ*) of electroactive non-modified GOx in Nafion/GOx/OMC-COOH and GOx-NH_2_ in Nafion/GOx-NH_2_/OMC-COOH was 2.41 × 10^−9^ mol·cm^−2^ and 2.91 × 10^−9^ mol·cm^−2^, respectively. These values are far greater than that of monolayer GOx covering the surface of the bare electrode (2.86 × 10^−12^ mol·cm^−2^) [74], indicating that the enzyme achieves a multilayer and three-dimensional coverage of OMC-COOH. After calculation, the enzyme surface coverage of the Nafion/GOx-NH_2_/OMC-COOH electrode was 20.72% higher than that of the Nafion/GOx/OMC-COOH electrode. In addition, the surface coverage of the Nafion/GOx-NH_2_/OMC-COOH electrode was wider than that of GOx coated on the surface of nickel oxide/hydroxide (NiO/Ni(OH)_2_) (1.43 × 10^−10^ mol·cm^−2^) [75], Nafion/GOx-CoS-MWCNTs/GCE (2.97 × 10^−11^ mol·cm^−2^) [76], and Nafion/GOx/ERGO-PLL/GCE (1.64 × 10^−11^ mol·cm^−2^) electrodes [4]. These calculation results show that, compared to the Nafion/GOx/OMC-COOH and other electrodes, the Nafion/GOx-NH_2_/OMC-COOH electrode has an advantage in effective electron transfer [73]. Above all, the amination treatment could increase the enzyme surface coverage and, thus, improve the effective electron transfer efficiency for the bioelectrode.

The work in this paper and the work in other references have been compared, the results of which are shown in Table 2. Compared with other bioelectrodes constructed with GOx, our electrode exhibits a higher current, and the GOx surface coverage and apparent electron transfer rate constant are higher than those of other electrodes. The results show that the chemical amination of enzymes can increase the enzyme loading of the bioelectrode, accelerate the electron transfer rate, and achieve the purpose of improving electrode performance.

## 5. Conclusions

Amination could be applied as a promising modification method for constructing effective glucose oxidase bioelectrodes, because it allows the enzyme to be more stable and efficiently immobilized on the mesoporous carbon material activated with carboxyl groups. In comparison to the non-modified enzyme, amination and the immobilization process can improve the electrochemical properties of the bioelectrode, such as its apparent electron transfer rate constant and charge-transfer coefficient.

## Figures and Tables

**Table 1:** Enzyme activity of free enzyme or enzyme immobilized in COOH-OMC.

Type	Quantity of the Immobilized Enzyme (GOx mg/OMC g)	Immobilized Enzyme Activity (U/g)	Unit Free Enzyme Activity (U/mg)
GOx	/	/	417.74 ± 22.22
GOx-NH_2_	/	/	364.41 ± 4.44
GOx/OMC-COOH	44.72 ± 0.88	58,290.47 ± 3055.42	1303.52 ± 68.33
GOx-NH_2_/OMC-COOH	124.01 ± 1.49	67,956.17 ± 4687.75	547.97 ± 37.80

**Table 2:** Comparison between various GOx bioelectrodes.

Electrode	Anodic Peak Current	Reduction Peak Current	GOx Surface Coverage (*Γ*)	*k_s_*	References
Nafion/GOx-NH_2_/OMC-COOH	0.324 mA	−0.394 mA	2.91 × 10^−9^ mol·cm^−2^	2.54 s^−1^	This study
GC/TiO_2_-IOSL/GOD	0.020 mA	−0.022 mA	1.23 × 10^−9^ mol·cm^−2^	1.74 s^−1^	[77]
RGO-AuNPs/PNR/GOx	0.006 mA	−0.012 mA	3.06 × 10^−9^ mol·cm^−11^	1.73 s^−1^	[59]
GOD/HRP/Ag/CNT/ITO	0.022 mA	−0.008 mA	3.52 × 10^−10^ mol·cm^−2^	1.76 s^−1^	[78]
GCE/MnO_2_ -G/PTA/Frt/GOx	0.120 mA	−0.180 mA	9.7 × 10^−10^ mol·cm^−2^	1.96 s^−1^	[79]

## Data Availability

The data presented in this study are contained within the article.
